# Estimating the abundance of airborne pollen and fungal spores at variable elevations using an aircraft: how high can they fly?

**DOI:** 10.1038/srep44535

**Published:** 2017-03-16

**Authors:** Athanasios Damialis, Evangelos Kaimakamis, Maria Konoglou, Ioannis Akritidis, Claudia Traidl-Hoffmann, Dimitrios Gioulekas

**Affiliations:** 1 Chair and Institute of Environmental Medicine, UNIKA-T, Technical University of Munich and Helmholtz Zentrum München, Germany - German Research Center for Environmental Health, Augsburg, Germany; 2 CK-CARE, Christine Kühne – Center for Allergy and Research and Education, Davos, Switzerland; 3 Department of Ecology, School of Biology, Aristotle University of Thessaloniki, Thessaloniki, Greece; 4 1st Pulmonary Department, “G. Papanikolaou” General Hospital of Thessaloniki, Thessaloniki, Greece; 5 Internal Medicine Department, “G. Gennimatas” General Hospital of Thessaloniki, Thessaloniki, Greece; 6 Pulmonary Department, Faculty of Medicine, Aristotle University of Thessaloniki, Thessaloniki, Greece

## Abstract

Airborne pollen and fungal spores are monitored mainly in highly populated, urban environments, for allergy prevention purposes. However, their sources can frequently be located outside cities’ fringes with more vegetation. So as to shed light to this paradox, we investigated the diversity and abundance of airborne pollen and fungal spores at various environmental regimes. We monitored pollen and spores using an aircraft and a car, at elevations from sea level to 2,000 m above ground, in the region of Thesssaloniki, Greece. We found a total of 24 pollen types and more than 15 spore types. Pollen and spores were detected throughout the elevational transect. Lower elevations exhibited higher pollen concentrations in only half of plant taxa and higher fungal spore concentrations in only *Ustilago*. Pinaceae and *Quercus* pollen were the most abundant recorded by airplane (>54% of the total). Poaceae pollen were the most abundant via car measurements (>77% of the total). *Cladosporium* and *Alternaria* spores were the most abundant in all cases (aircraft: >69% and >17%, car: >45% and >27%, respectively). We conclude that pollen and fungal spores can be diverse and abundant even outside the main source area, evidently because of long-distance transport incidents.

Allergic diseases in industrialized countries, including asthma, have increased in incidence and severity^1^,^2^,^3^,^4^. This is partially attributed to the increased concentration of air pollutants and aeroallergens (pollen and fungal spores)^1^,^5^. Various epidemiological studies in western countries report frequencies of allergic rhinitis and asthma of around 30–40%, even 50% for children^3^,^6^,^7^,^8^,^9^. It is also known that aeroallergens are responsible for a large percentage of bronchial asthma exacerbations in children and adolescents as well as increased hospitalizations^10^, whilst recent studies raise the percentage of allergic predisposition to aeroallergens in patients with mild to moderate asthma up to 90–95%^11^. Also, many researchers have demonstrated increasing positive correlations between aeroallergen concentrations and symptoms of allergic rhinitis and asthma in patients with previous sensitization^12^,^13^.

The above changes have been frequently attributed to climate change; nonetheless, most studies refer to urban and highly populated areas and, hence, diversity and abundance variability of pollen and fungal spores in the atmosphere remain a controversial issue and uninterrupted measurements of their concentrations could possibly shed some light to this relationship. Studies have shown that different meteorological factors (especially air temperature, relative humidity, presence of storms and wind vectors) most probably alter the distribution of airborne pollen and fungal spores into space and time, causing variable respiratory problems in chronic respiratory allergy patients^5^,^10^. Cecchi and colleagues^14^ have strongly recommended the continuous monitoring of these allergenic agents and a state of alertness for proper countermeasures in cases of high concentrations in the air.

Previous studies have tried to quantify the distribution of various pollen types’ abundances in the atmosphere for certain periods of time^15^,^16^. This is also true for the study area, Thessaloniki, Greece, resulting in distribution calendars of both fungal spores and pollen, based on long-term records^17^,^18^. As de Linares and colleagues^19^ have documented, allergen content in the atmosphere is positively correlated with airborne pollen concentrations and, noticeably, regardless of the values of any meteorological factor. Hence, it is really important to monitor airborne allergenic pollen and fungal spores and evaluate their abundance and, concomitantly, the associated health risk. However, up to date pollen records refer to near-ground-level measurements. Thus, the corresponding distribution of pollen and fungal spores at higher elevations above ground level has been attempted only scarcely, as in the case of Raynor and colleagues^20^, who measured *Ambrosia* pollen at variable heights on a meteorological tower of 108 m maximum height. They found that the diversity in size, distance, and location of ragweed sources do not necessarily correlate with the timing and magnitude of pollen emission; this is partly due to the increased variability in meteorological conditions between source and sampling locations, which overall constitute a complex set of hard to measure variables at the sampling site. Comtois and colleagues^21^ counted pollen up to 600 m using an air tethered balloon. In their research, pollen were observed at all elevations with a tendency towards higher pollen concentrations at greater elevations. Likewise, Gruber and colleagues^22^ measured air particles using several means (among which an aircraft) and found that pollen can be found up to 2,000 m. On the other hand, even small-scale variations in sampling height (i.e. of just 5 m) were found to significantly differentiate measurements of aeroallergens, like pollen and fungal spores^23^,^24^,^25^,^26^,^27^,^28^. However, this variability was frequently affected by year-to-year fluctuations in meteorological variables, local vegetation and by the fungal or plant taxon under study.

The above already provide an indication of the capability of airborne pollen and fungal spores to travel great distances from the original source of production under favourable environmental conditions, mainly wind components and lack of rainfall^29^. Hence, phenomena of long- or medium- range transport are quite frequent^30^. Such long-distance transport incidents have been observed in the area of Thessaloniki for pollen grains from several plant taxa (at a sampling height of approximately 35 m above ground), and hence they can cause respiratory allergy symptoms, even outside the main flowering period of the respective plant species^29^. This has been documented in other areas as well, as in Spain by Feo Brito and colleagues^13^, who proved that allergenic activity of grasses and relevant symptoms can exist throughout the year, even outside the pollen season, posing an important health risk for allergic individuals.

Most of the above studies refer to airborne pollen. Relevant research on fungal spores is far too limited^25^. In terms of allergic symptoms, Crameri and colleagues^31^ have pointed out that fungi, compared to pollen, are a neglected and underestimated source of respiratory allergy. Nonetheless, it is widely known that fungal spores frequently induce respiratory allergy symptoms in children, manifested even as acute respiratory failure^32^,^33^. In terms of changing biodiversity, Berman^34^ expressed concerns about how ongoing climate change can alter biodiversity and, in turn, influence allergic sensitisation. Beggs^35^ has also reviewed that there is urgent need to examine air particles other than pollen, particularly when so little information is available.

The aim of the present study was to assess the circulation patterns of airborne pollen and fungal spores, of all detected taxa, in space (at horizontal and also vertical scale) and time. This was attempted not only at the standard height that allergenic pollen and fungal spores are usually measured, but at variable elevations, so as to simulate pollen and fungal-spore vertical movement and also deposition. Thus, to our knowledge, this research is the first of its kind worldwide, since all previous studies rarely deal with long-distance transport phenomena for a wide spectrum of taxa, especially of fungal spores, and at high above-ground elevations. Our original hypothesis was that we would be able to identify isolated incidents of higher concentrations of pollen and spores at higher above ground elevations, at least for some plant and fungal taxa. To achieve the above, an appropriate methodology was developed for the sampling of aeroallergens and the quantification and analysis of aerobiological data.

## Results

### Airborne pollen

A total of 24 pollen types were recorded from aircraft and car samples and at variable heights (Table 1). In terms of diversity, pollen from 23 different taxa were recorded by airplane, compared to 15 recorded by car, at ground-level. By both sampling means, 10 pollen types exhibited a concentration higher than 0.5% of the total. This corresponded to 93.1% for aircraft sampling and 97.6% for car sampling (Table 1). Interestingly, in airplane samples tree pollen were predominant by 54% (Pinaceae and *Quercus*), whereas in car samples Poaceae pollen type was by far the most abundant (78%). Records by car were significantly higher than those by aircraft in only 2 out of the 10 pollen types examined and noticeably both pollen types were from herbaceous taxa, Chenopodiaceae and Poaceae (Table 1). Pollen concentrations sampled on the aircraft were usually higher and this was particularly true for Pinaceae (Table 1).

This increase with aircraft sampling was higher than 1100% in *Olea* and 773% in Pinaceae. The only cases where concentrations of pollen were higher at lower elevation (car sampling) were in Poaceae (61%) and Chenopodiaceae (46%) (Table 1). For the period examined, the total pollen count at higher elevation was also much higher than that at ground level, with a difference of more than 70% (Table 1). Analysis of covariance displayed an overall tendency towards higher pollen concentrations at higher elevations (*p* < 0.001, *R*^2^ = 0.36). Additionally to the above, analysis of variance was further performed, including also the seasonality element (Calendar day as a weighted-covariate factor). As seen in Table 1, in only two cases, Chenopodiaceae and Poaceae pollen, aircraft records exhibited lower records than ground-level records. However, Cupressaceae, *Quercus* and *Thalictrum* pollen proved not to be significantly changing with increasing elevation, even though they tended to present higher pollen concentrations with aircraft sampling (Table 1).

Attempting to model airborne pollen levels at different elevations and habitats, pollen concentrations were regressed against habitat type and elevation (GLM, full factorial regression). In all cases, significant relationships were found for at least one of the examined independent variables. The significance levels, coefficients of determination and partial correlations of each variable (so as to study individual effects) are given in Table 2. It was observed that only in half of the studied taxa pollen concentrations were higher in urban environments and low elevation; no particular pattern was found in pollen concentration abundance, related to elevation, habitat type, herbaceous versus woody plants or interactions between them (Table 2). The strongest correlations with habitat type and elevation were observed in woody taxa, namely of Cupressaceae, Pinaceae, Poaceae and *Quercus*, which all exhibited coefficients of determination higher than 0.50; these were inverse in the first three cases and positive only in the case of *Quercus* (Table 2).

**Figure 1:**
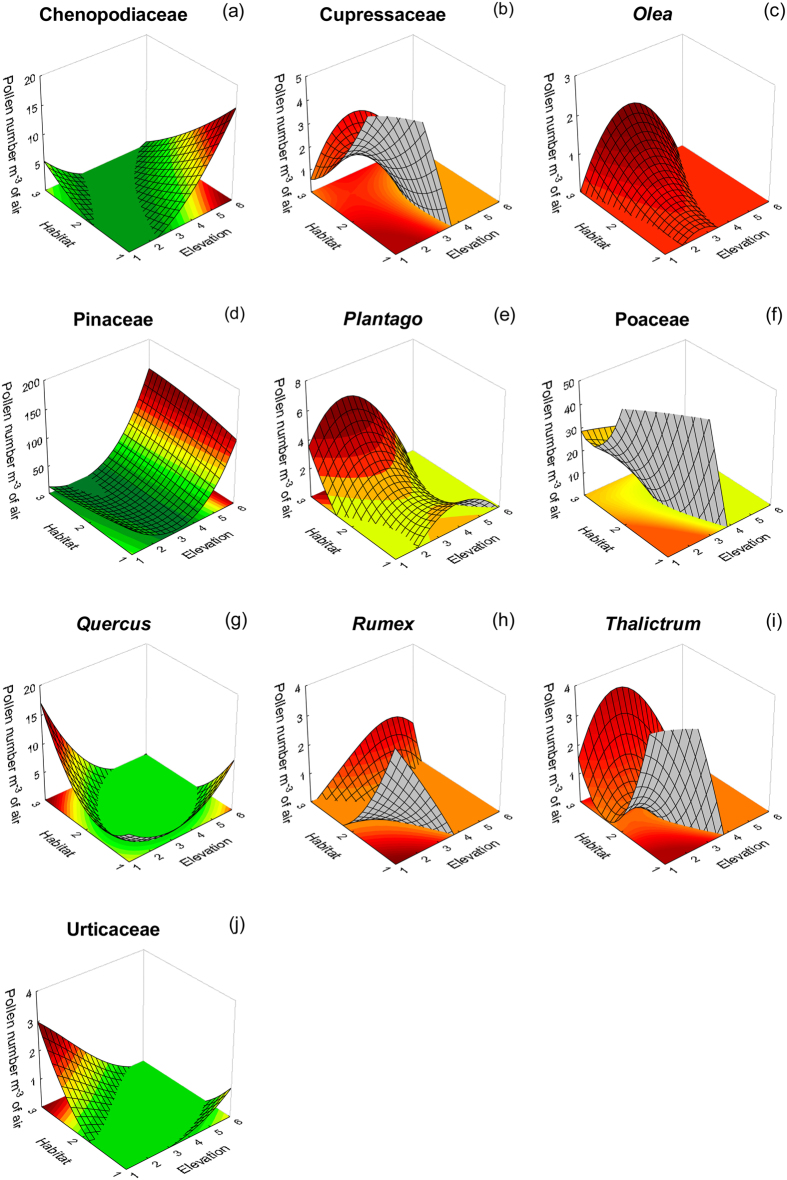
Factorial regression plots of airborne pollen concentrations per cubic metre of air (dependent variable) for 10 taxa (Fig. 1(a–j)), against habitat type (urban environment, semi-urban and natural ecosystem) and elevation (0–2000 m). Habitat codes: 1: urban environment, 2: semi-urban environment, 3: natural environment. Elevation codes: 1: lower than 300 m, 2: 301–600 m, 3: 601–900 m, 4: 901–1200 m, 5: 1201–1500 m, 6: higher than 1500 m. Spline fitting has been applied in all cases (curve stiffness equals to 0.5).

Factorial regression analysis and posthoc tests after factorial ANOVA revealed pollen circulation patterns based mainly on the interaction effects of both elevation and habitat type, rather than the individual effects of either of the two studied parameters (Fig. 1). For example, Pinaceae pollen were more abundant at high and natural locations (Fig. 1(d)). On the contrary, Cupressaceae pollen were observed more frequently at lower and urban environments (Fig. 1(b)) and *Quercus* pollen at lower and natural environments (Fig. 1(g)). Only few taxa exhibited higher pollen concentrations’ correlations with the individual effects of examined parameters, like in the case of Poaceae, whose pollen were more abundant at lower elevations or at natural habitats (Fig. 1(f)).

### Airborne fungal spores

A total of 19 fungal spore types were identified from aircraft and car samples and at variable elevations (Table 3). In terms of diversity, spores from more than 15 different fungal taxa were recorded by airplane, compared to only 8 recorded by car, at ground-level. By both sampling means, seven spore types exhibited a concentration higher than 0.5% of the total. This corresponded to 93.6% for aircraft sampling and 99.7% for car sampling (Table 3). Interestingly, the most predominant spore types were those of *Cladosporium* and *Alternaria*, in both airplane and car samples (87% and 73%, respectively). Many fungal taxa were not observed at all at ground-level measurements, like those of Myxomycetes, *Arthrinium* etc. (Table 3). So for the period examined, the total fungal spore count at higher elevation was slightly higher than that at ground level, with a difference of 13% (Table 3). Of the 14 different spore types identified, only four displayed lower airborne concentrations at higher elevations (Table 3).

Records by car were significantly higher than those by aircraft in three out of the seven fungal spore types examined, specifically in *Alternaria*, Ascospores and *Ustilago* (Table 3). This increase with aircraft sampling was as high as 73% in the case of *Cladosporium* (Table 3). Of the total number of fungal types identified during the study period, only in 4 fungal taxa spores were higher in ground-level samples (Table 3). *Ustilago* (93%) and *Alternaria* (29%) displayed the greater differences against aircraft samples at higher elevations (Table 3). Overall, for the period examined, the total spore count at higher elevation was higher than that at ground level, with a difference of 13% (Table 3). Analysis of covariance displayed an increased variance and no significant differences in spore concentrations between higher and lower elevations (*p* > 0.05, *R*^2^ = 0.19).

Attempting to model airborne fungal spore levels at different elevations and habitats, spore concentrations were regressed against habitat type and elevation (GLM, full factorial regression). In all cases, significant relationships were found for at least one of the examined independent variables. The significance levels, coefficients of determination and partial correlations of each variable (so as to study individual effects) are given in Table 4. It was observed that in only one case (*Ustilago*) spore concentrations were higher in urban environments and low elevation; it seems that most fungal taxa exhibit higher spore concentrations at higher elevations and at natural environments (Table 4). The strongest correlations with either habitat type or elevation were observed in *Alternaria*, Ascospores, *Cladosporium* and *Ustilago*, although their coefficients of determination were in all cases rather low; these relationships were positive in the first three cases and inverse only in the case of *Ustilago* (Table 4).

**Figure 2:**
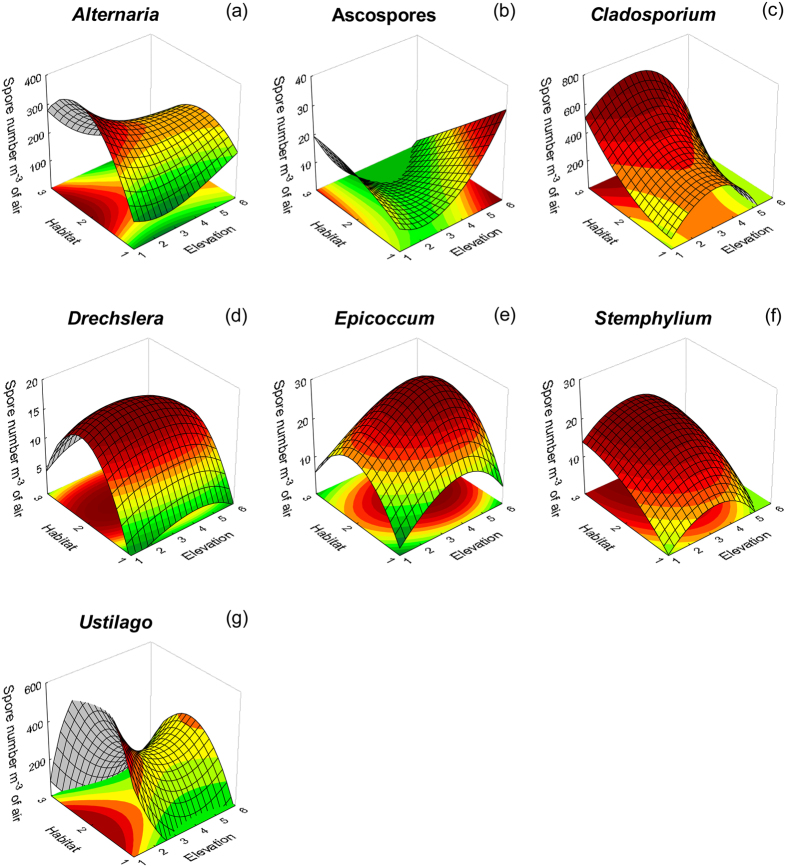
Factorial regression plots of airborne fungal spore concentrations per cubic metre of air (dependent variable) for 7 taxa (Fig. 2(a–g)), against habitat type (urban environment, semi-urban and natural ecosystem) and elevation (0–2000 m). Habitat codes: 1: urban environment, 2: semi-urban environment, 3: natural environment. Elevation codes: 1: lower than 300 m, 2: 301–600 m, 3: 601–900 m, 4: 901–1200 m, 5: 1201–1500 m, 6: higher than 1500 m. Spline fitting has been applied in all cases (curve stiffness equals to 0.5).

Factorial regression analysis and posthoc tests after factorial ANOVA revealed fungal spore circulation patterns based mainly on the interaction effects of both elevation and habitat type, rather than the individual effects of either of the two studied parameters (Fig. 2). For example, *Cladosporium, Epicoccum* and *Stemphylium* spores were more abundant at high and natural locations (Fig. 2(c,e and f)), respectively). On the contrary, *Ustilago* and *Alternaria* showed higher spore concentrations at lower elevations and mainly natural environments (Fig. 2(a,g)). Interestingly, very few fungal taxa exhibited higher spore concentrations’ correlations with the individual effects of examined parameters, like in the case of *Drechslera*, whose spores were more abundant at semi-urban environments and regardless of the elevation examined (Fig. 2(d)).

## Discussion

This research is among the very first worldwide to deal with both vertical and horizontal transportation of airborne pollen and fungal spores and for a wide spectrum of taxa. Its usefulness is pronounced both from the conservation and from the public health perspective: pollen and spores can be detected in a diverse range of environments, frequently independent of the regional sources. This was concluded from the examination of airborne pollen and spores’ measurements taken from both ground level and various heights using an aircraft, under various environmental regimes. Our original hypothesis was that the majority of detected pollen and spores in the atmosphere would originate from near-ground sources: this was correct in certain cases, like Poaceae pollen at ground-level measurements. We also hypothesised that there may be only isolated incidents of increased pollen and spore exposure: this has been only partly validated, as aeroallergens are actually more frequently observed at higher elevations, 2,000 m or higher. Lack of safe periods or locations for allergic sufferers has been reported previously by various researchers^36^.

The above comprise an indication that pollen and fungal spores can be transported in the atmosphere to a greater than originally thought distance, under suitable weather conditions. It is generally accepted that the majority of sampled air particles comes from local sources^37^. Nevertheless, pollen from taxa not representative of the local or regional vegetation have been frequently detected, implying long-range transport. For example, it has been reported that pollen can be transferred above oceans or among continents and at extreme environments, covering distances up to hundreds or thousands of kilometers away from the source^38^,^39^,^40^,^41^,^42^,^43^,^44^. To our knowledge, there is limited information on fungal spores^45^.

The above scenario of medium- to long- range transport of pollen and fungal spores gets even stronger if we also consider that the findings of the current study reveal a higher diversity of both pollen and spores at higher elevations. One could claim that this could be the result of vertical movement of ground-level sources of examined particles, but our data and the statistical analysis have actually proved frequently lower concentrations at lower elevations. Consequently, the most probable scenario is that pollen and fungal spores are mainly found at higher elevations due to long-range transport or intense re-suspension phenomena.

According to pollen aerobiological data from the metropolitan city of Thessaloniki, which is close to the present study area, for the period 1987–2005^29^, long- and medium- range pollen transport incidents have been taking place regularly. In fact, 11 taxa (out of 16) contribute to the total more than 1%, and 7 taxa more than 85% to the total^46^. These are namely Cupressaceae (23.2%), *Quercus* (20.8%), Urticaceae (10.8%), Pinaceae (10.1%), Oleaceae (7.6%), *Platanus* (6.7%) and Poaceae (6.0%). According to spore aerobiological data from Thessaloniki for the period 1987–2005^47^, 7 taxa (out of 14) contribute to the total more than 1%, and 3 taxa more than 90% to the total, namely *Cladosporium* (72.0%), *Alternaria* (10.1%) and *Ustilago* (8.0%).

It is quite interesting that in biodiversity terms aerobiological monitoring can yield different results from the aircraft sampling findings showed here. In the case of fungal spores, it is remarkable that spores from the same fungal taxa are always detected regardless of the monitoring method. This implies that the prevailing spores from *Cladosporium, Alternaria* and *Ustilago* can derive from the dominance of these fungal species in the local and regional mycoflora. Also, the above may be due to the ability of these genera to either be highly competent against other fungal species or be capable to produce vast numbers of spores. *Alternaria*’s high competitive capacity and *Cladosporium*’s high sporulation rate have already been documented^48^. Noticeably, *Cladosporium* spores have been even detected in Antarctica^42^.

On the other hand, in the case of pollen, the patterns are more nuanced. There is no clear parallel pattern between aerobiological results at lower elevation compared to results from aircraft sampling at higher elevations. What is common is that woody plants seem to be always more abundantly represented, i.e. Cupressaceae, Pinaceae, *Quercus*. This could be either to higher participation of specific species in local vegetation or that woody plants may present higher pollen production compared to herbaceous plants, i.e. Poaceae. In terms of absolute numbers this may be true^49^,^50^,^51^, however, the combination of these factors ought to be also examined.

The present study showcases the complexity of the atmospheric circulation of pollen and fungal spores. The original hypothesis claimed that elevation would play a role for airborne pollen and spore concentration levels, but this was not quite accurate: it was frequently noted that neither habitat type alone nor elevation alone were able to sufficiently explain the concentration levels of pollen and fungal spores. Relationships were non-linear and usually the interaction of these two factors was the decisive factor for forecasting airborne concentrations. Therefore, the general conclusion reached was that pollen and fungal spores reflect regional vegetation, but elevation can play a synergistic or antagonistic role, depending on the existence of long-distance transport. The complexity of the dispersion and atmospheric circulation of such microorganisms has been pointed out also in a review by Pearce and colleagues^42^, who have indicated a wide variety of potential factors affecting the dispersion of fungal spores and pollen, especially in long-distance transport incidents.

Particularly in the case of fungal spores, even the interaction effects of habitat type and elevation provided only a rough approximation of spore concentrations, potentially as the result of the numerous sources of fungal species. Most fungal species preferred natural environments and high elevations, except for *Alternaria* and *Ustilago*, which preferred lower elevations as they comprise notorious phytopathogens, particularly in cereal crops^52^.

Regarding pollen, there were cases that accurately reflected the regional vegetation, like in Cupressaceae pollen (increasing at lower and urban environments) and *Quercus* pollen (increasing at lower and natural environments). But in other cases, like in Pinaceae, pollen were most abundant at high elevations and natural habitats, which supports the long-distance transport scenario. Other researchers have also documented long-distance transport of Pinaceae pollen, probably because of its aerodynamics^38^.

This study was among the first to assess the airborne pollen and fungal spore levels at various elevations and to correlate them with habitat types. As aircraft sampling requires higher financial and technical support, it was admittedly not feasible to perform as frequent samplings as wished for. Unmanned aircraft sampling^53^,^54^ would potentially resolve this issue, although they have many limitations concerning the maximum elevation capacity or air traffic and safety issues. Moreover, the next step would be to attempt correlating airborne pollen and spore levels at higher elevations with allergic patients’ symptoms. Thus, more timely forecasts of allergy-risk periods may be elaborated or we may be able to detect seasons and areas with increased risk for symptoms even outside the main pollen or spore season as recorded by urban biomonitoring stations.

## Conclusion

It is concluded that there is increased risk of pollen and fungal spore exposure regardless of the elevation of sampling. It was found that airborne pollen and fungal spores can be frequently distributed far away from the main emission sources, as indicated by high aboveground measurements. Hence, questions are raised considering A) the safety of previously thought as harmless environments and B) the increased possibility of aeroallergens being actually found in a more diverse range of environments. Biodiversity tends to be higher at higher elevations, an indication also of long-distance pollen and fungal spore transport. Abundance, likewise, tends to be higher, as pollen and spores frequently exhibit higher concentrations at higher elevations. Therefore, it is comprehended that there is a great allergenic potential at higher elevations. This, to an extent, is representative of the local vegetation, conditions and sources of pollen and fungal spores. Hence, it is suggested that towards timely informing of allergic patients, additional and complementary biomonitoring methods need to be followed, like airplane aeroallergen sampling, which allows for more generalized pollen and spore abundance estimations, including also medium- and long-range transport of aeroallergens.

## Material and Methods

### Study area

The study was conducted in the region of Thessaloniki prefecture, northern Greece. The city of Thessaloniki is located to the north of Thermaikos Gulf, in the Aegean Sea, with a Mediterranean climate. Several forests grow close, mainly of pines, cypresses and oaks^55^,^56^. Within the city, poplars, planes, olives, cypresses and pines grow in parks; regarding herbaceous plants, many grasses, amaranths, goosefoots, wall pellitories and nettles can also be found in abundance^57^. Farther away, cereal crops along with wild grasses also exist, together with poplar, hazel and olive plantations^58^,^59^.

### Aerobiological sampling

Aeroallergen sampling extended from March to July 2010, which has been reported as the main pollen and fungal spore season for the majority of most abundantly represented taxa in Thessaloniki^17^,^18^. Aeroallergens were sampled on the northern fringes of the city every 10–15 days, depending on the weather (days with no heavy precipitation or strong winds). In total, 72 samples were taken from nine flights, not including those taken for the standardization of the method.

Sampling took place using two different transportation means, viz. a two-seat Cessna 152 aircraft and a car. Sampling routes were followed at the same time so as to obtain comparable results. Samples by use of the aircraft were also taken at different heights: in particular, this type of aircraft has the ability to fly at elevations of up to 3,600 meters above sea level and at speeds between 70–120 km h^−1^. The car sampling followed the route of the ring road of the city, peripherally to the greater city boundaries. For both transportation means, two people were always on board, the pilot/driver and a researcher taking the aeroallergen samples. So as to check specific effects, we also measured the flight elevation (200–2,000 m), sampling direction (octants) and airplane and car speed (km h^−1^). Sampling took place always at the same period of the day (early afternoon).

Microscope slides, coated with adhesive substance (Burkard gelvatol), were exposed to open air out of the aircraft and car window, for two minutes and for each selected locality (vegetation type). Airborne particles (among which pollen grains and fungal spores) that were captured onto slides, were then covered with cover slips after adding a solution of gelatine-glycerol-phenol-saffranine. All aeroallergens were identified and counted under light microscope at magnifications of x600 and x400, for fungal spores and pollen respectively. The whole slide area was examined. Aeroallergen concentrations were expressed as numbers of spores or pollen per m^3^ of air, per taxon and for each given sampling day. The latter was achieved by additionally measuring the aircraft’s flying and car’s running speeds. Thus, we were able to extrapolate from the volume of the sample taken [(*surface of the slide*) x (*distance covered with steady speed*)] to volume unit, viz. 1 m^3^ of air. The extrapolation is based on the same technique used for estimating pollen and fungal spore counts measured by Hirst-type volumetric traps^60^ and this was followed so as to have comparable results with the traditional aeroallergen monitoring methods. Sampling was held at predetermined flight elevations from 200 to 2,000 meters above sea level.

So as to decide on the optimum method to apply for pollen sampling, we checked for (A) the placements of the slide during flight: closer to and farther from the engine, upper and lower from it. Thus, the slide exposure distance from the aircraft window was also tested (researcher’s arm length). No significant differences were found between slide placements, including both distance from the aircraft engine or window (*p* > 0.05, ANOVA). The selected position, however, was as far from the window and engine as possible.

(B) Sampling intervals: 1, 2, 3 and 4 minutes. Taking into account the aircraft speed and wishing to reduce the turbulence effect as much as possible, firstly we kept speed to a minimum (and so as to be comparable with the car speed). Secondly, we found that sampling time was not a significant factor (*p* > 0.05, ANOVA), taking also into account the relatively higher air volume sampled because of the speed of the aircraft. For example, for an average speed of 80 km h^−1^, 3.3 m^3^ of air would be sampled on a microscope slide within two minutes, which is actually a quite large volume of air. Of this, we identified and counted all pollen and spores from all the sampling area of each slide. If compared to the volumetric biomonitoring technique, per bi-hourly interval from each daily slide of a Hirst-type sampler, we normally examine one vertical transect, which in our case (width of microscope lens) is 0.485 mm. This corresponds to 12.1% of the total 4 mm of the bi-hourly sample, and this, in terms of sampling time corresponds to 14.5 minutes. Given the volume sampled for the same interval via aircraft, the 2-minute interval seems appropriate. Regarding potential turbulence and the actual possibility to capture different pollen and spore types within this time interval and with the given aircraft speed, indeed this was something that is not avowedly easy to control. Nonetheless, the findings obtained already indicate that even smaller-sized particles, i.e. Urticaceae pollen and *Cladosporium* spores are captured and, noticeably, in large amounts. But even if there is some kind of bias because of increased turbulence under certain conditions, this would expectedly lead to decreased concentrations of specific taxa, which is not the case here.

### Data analysis

Abundance patterns were investigated for both pollen and fungal spores. All data were processed as raw concentrations of aeroallergens per cubic metre of air, but natural logarithms were also tested. Statistical analysis was performed for those taxa whose pollen and fungal spores contributed more than 0.5% to the total pollen and spore concentration, respectively, for both car and aircraft sampling. Differences were investigated between pollen records by aircraft and car (t test and one-way ANOVA) and also in atmospheric pollen concentrations among sampling localities (urban vs. semi-urban vs. natural environment, categorised from 1 to 3 (1: urban environment, 2: semi-urban environment, 3: natural environment)), elevation (0–2,000 m, categorised from 1 to 6 (1: lower than 300 m, 2: 301–600 m, 3: 601–900 m, 4: 901–1200 m, 5: 1201–1500 m, 6: higher than 1500 m)) and sampling direction (one-way and full-factorial ANOVA and ANCOVA). When significant differences were observed, potential relationships between variables were examined with factorial regressions and spline fitting was applied in all cases (curve stiffness equal to 0.5 was applied, so as to check for the overall pattern, instead of particularities). Residual analysis for each dataset provided an assessment of the remaining noise. All statistical analyses were performed using Statistica software.

## Additional Information

**How to cite this article:** Damialis, A. *et al*. Estimating the abundance of airborne pollen and fungal spores at variable elevations using an aircraft: how high can they fly? *Sci. Rep.* **7**, 44535; doi: 10.1038/srep44535 (2017).

**Publisher's note:** Springer Nature remains neutral with regard to jurisdictional claims in published maps and institutional affiliations.

## Figures and Tables

**Table 1:** Average pollen concentrations (m^−3^ of air) by aircraft and car (total of 72 samples from 9 dates).

Taxon	Aircraft records		Car records		% Change between sampling means (*p*)
	Average concentration (pollen m^−3^ of air)	Contribution to the total concentration	Average concentration (pollen m^−3^ of air)	Contribution to the total concentration	
Pinaceae	123 ± 36	28.4%	14 ± 5	5.6%	+773% (*)
*Quercus*	112 ± 35	25.9%	9 ± 5	3.5%	+1184% (ns)
Poaceae	76 ± 16	17.6%	196 ± 56	77.7%	−61% (***)
*Plantago*	22 ± 8	5.0%	11 ± 7	4.5%	+89% (ns)
*Olea*	18 ± 9	4.2%	1 ± 1	0.6%	+1184% (*)
Cupressaceae	17 ± 7	3.8%	3 ± 3	1.1%	+520% (ns)
*Thalictrum*	14 ± 6	3.3%	3 ± 3	1.0%	+442% (ns)
Urticaceae	13 ± 8	3.0%	2 ± 2	1.0%	+437% (**)
Oleaceae	7	1.6%	0	0.0%	n/a
*Fagus*	6	1.4%	1	0.3%	+765%
*Rumex*	6 ± 2	1.3%	2 ± 2	0.7%	+232% (*)
*Carpinus*	4	0.9%	0	0.0%	n/a
Chenopodiaceae	3 ± 2	0.7%	5 ± 6	2.1%	−46% (***)
Typhaceae	2	0.5%	0	0.0%	n/a
Rosaceae	2	0.5%	0	0.0%	n/a
Apiaceae	1	0.3%	0	0.0%	n/a
*Tilia*	1	0.3%	0	0.0%	n/a
*Ulmus*	1	0.3%	0	0.0%	n/a
*Platanus*	1	0.3%	0	0.0%	n/a
Asteroideae	1	0.2%	1	0.3%	+42%
Cichorioideae	1	0.1%	2	0.6%	−67%
Juncaceae	1	0.1%	0	0.0%	n/a
Myrtaceae	1	0.1%	1	0.6%	−65%
Cyperaceae	0	0.1%	2	0.7%	−76%
****Total****	**433**	100.0%	**252**	100.0%	+**72%**

Contributions (%) to the total pollen amount are given per taxon (in descending order for the aircraft records), along with differences between sampling means (in parenthesis results from one-way, weighted-moments ANOVA is given, ***p < 0.001, **p < 0.01, *p < 0.05, ns: not significant relationship).

Statistical analysis was performed for those taxa whose pollen contributed more than 0.5% to the total pollen concentration, respectively, for *both* car and aircraft sampling.

**Table 2:** Factorial Regression results of pollen concentrations (m^−3^ of air) for 10 taxa, against habitat type (urban environment, semi-urban and natural ecosystem), elevation (0–2000 m) and their interaction effect.

Plant taxon	*p*	*R*^2^	Partial correlations		
			Habitat type (1)	Elevation (2)	(1)*(2)
Chenopodiaceae	0.001	0.21	+0.42	+0.36	−0.41
Cupressaceae	0.001	0.61	−0.75	−0.73	+0.70
*Olea*	0.001	0.07	+0.24	ns	ns
Pinaceae	0.001	0.52	−0.29	−0.25	+0.42
*Plantago*	0.001	0.29	+0.47	+0.32	−0.37
Poaceae	0.001	0.61	−0.73	−0.71	+0.67
*Quercus*	0.001	0.55	+0.49	+0.18	−0.36
*Rumex*	0.001	0.23	−0.46	−0.38	+0.42
*Thalictrum*	0.001	0.14	−0.30	−0.31	+0.27
Urticaceae	0.001	0.50	+0.50	+0.21	−0.34

Partial correlation coefficients, *p* and *R*^2^ are given.

For partial correlations, “+” indicates a positive correlation, “−” a negative correlation; “ns” indicates a non-significant relationship (*p* > 0.05).

**Table 3:** Average fungal spore concentrations (m^−3^ of air) by aircraft and car (total of 72 samples from 9 dates).

Taxon	Aircraft records		Car records		% Change between sampling means (*p*)
	Average concentration (spores m^−3^ of air)	Contribution to the total concentration	Average concentration (spores m^−3^ of air)	Contribution to the total concentration	
*Cladosporium*	1862 ± 523	69.6%	1074 ± 397	45.3%	+73% (***)
*Alternaria*	462 ± 139	17.3%	655 ± 340	27.6%	−29% (***)
*Stemphylium*	50 ± 22	1.9%	39 ± 17	1.7%	+27% (***)
Myxomycetes	41	1.5%	0	0.0%	n/a
*Epicoccum*	39 ± 16	1.5%	25 ± 11	1.1%	+57% (***)
*Ustilago*	36 ± 17	1.3%	505 ± 655	21.3%	−93% (***)
*Asperisporium*	36	1.3%	0	0.0%	n/a
Ascospores	30 ± 17	1.1%	41 ± 32	1.7%	−28% (***)
*Arthrinium*	29	1.1%	0	0.0%	n/a
*Drechslera*	25 ± 12	0.9%	23 ± 11	1.0%	+6% (ns)
*Gliomastix*	6	0.2%	0	0.0%	n/a
*Torula*	5	0.2%	0	0.0%	n/a
*Pleospora*	2	0.1%	7	0.3%	−46%
Other species	51	2.0%	0	0.0%	n/a
****Total****	**2674**	100.0%	**2369**	100%	+**13%**

Contributions (%) to the total spore amount are given per taxon (in descending order for the aircraft records), along with differences between sampling means (in parenthesis results from one-way, weighted-moments ANOVA is given, ***p < 0.001, **p < 0.01, *p < 0.05, ns: not significant relationship).

Statistical analysis was performed for those taxa whose fungal spores contributed more than 0.5% to the total spore concentration, respectively, for *both* car and aircraft sampling.

**Table 4:** Factorial Regression results of fungal spore concentrations (m^−3^ of air) for 7 taxa, against habitat type (urban environment, semi-urban and natural ecosystem), elevation (0–2000 m) and their interaction effect.

Fungal taxon	*p*	*R*^2^	Partial correlations		
			Habitat type (1)	Elevation (2)	(1)*(2)
*Alternaria*	0.001	0.07	+0.09	ns	−0.04
Ascospores	0.001	0.06	+0.11	+0.06	−0.11
*Cladosporium*	0.001	0.05	+0.14	+0.06	−0.06
*Drechslera*	0.001	0.01	+0.03	ns	ns
*Epicoccum*	0.001	0.02	ns	+0.07	−0.05
*Stemphylium*	0.001	0.03	+0.11	+0.04	−0.05
*Ustilago*	0.001	0.07	−0.20	−0.23	+0.20

Partial correlation coefficients, *p* and *R*^2^ are given.

For partial correlations, “+” indicates a positive correlation, “−” a negative correlation; “ns” indicates a non-significant relationship (*p* > 0.05).
